# Structure and lipid-binding properties of the kindlin-3 pleckstrin homology domain

**DOI:** 10.1042/BCJ20160791

**Published:** 2017-02-03

**Authors:** Tao Ni, Antreas C. Kalli, Fiona B. Naughton, Luke A. Yates, Omar Naneh, Mirijam Kozorog, Gregor Anderluh, Mark S.P. Sansom, Robert J.C. Gilbert

**Affiliations:** 1 Division of Structural Biology, Wellcome Trust Centre for Human Genetics, University of Oxford, Roosevelt Drive, Oxford OX3 7BN, U.K.; 2 Department of Biochemistry, University of Oxford, South Parks Road, Oxford OX1 3QU, U.K.; 3 Department for Molecular Biology and Nanobiotechnology, National Institute of Chemistry, Hajdrihova 19, 1000 Ljubljana, Slovenia

**Keywords:** lipid clustering, molecular dynamics, PH domain, surface plasmon resonance, X-ray crystallography

## Abstract

Kindlins co-activate integrins alongside talin. They possess, like talin, a FERM domain (4.1-erythrin–radixin–moiesin domain) comprising F0–F3 subdomains, but with a pleckstrin homology (PH) domain inserted in the F2 subdomain that enables membrane association. We present the crystal structure of murine kindlin-3 PH domain determined at a resolution of 2.23 Å and characterise its lipid binding using biophysical and computational approaches. Molecular dynamics simulations suggest flexibility in the PH domain loops connecting β-strands forming the putative phosphatidylinositol phosphate (PtdInsP)-binding site. Simulations with PtdInsP-containing bilayers reveal that the PH domain associates with PtdInsP molecules mainly via the positively charged surface presented by the β1–β2 loop and that it binds with somewhat higher affinity to PtdIns(3,4,5)P_3_ compared with PtdIns(4,5)P_2_. Surface plasmon resonance (SPR) with lipid headgroups immobilised and the PH domain as an analyte indicate affinities of 300 µM for PtdIns(3,4,5)P_3_ and 1 mM for PtdIns(4,5)P_2_. In contrast, SPR studies with an immobilised PH domain and lipid nanodiscs as the analyte show affinities of 0.40 µM for PtdIns(3,4,5)P_3_ and no affinity for PtdIns(4,5)P_2_ when the inositol phosphate constitutes 5% of the total lipids (∼5 molecules per nanodisc). Reducing the PtdIns(3,4,5)P_3_ composition to 1% abolishes nanodisc binding to the PH domain, as does site-directed mutagenesis of two lysines within the β1–β2 loop. Binding of PtdIns(3,4,5)P_3_ by a canonical PH domain, Grp1, is not similarly influenced by SPR experimental design. These data suggest a role for PtdIns(3,4,5)P_3_ clustering in the binding of some PH domains and not others, highlighting the importance of lipid mobility and clustering for the biophysical assessment of protein–membrane interactions.

## Introduction

Integrin-mediated adhesion between cells and the extracellular matrix is essential for the development of multicellular organisms. Integrins are heterodimeric (α and β) transmembrane receptors comprising a large extracellular domain for ligand binding (such as fibronectin, collagen, and vitronectin) and short cytoplasmic tails [1,2], and are responsible for bidirectional signal transduction across the cell membrane. The cytoplasmic tail of the β-subunit, despite its short length, is critical for integrin activation and has been shown to interact with several regulatory proteins. Talin was the first essential intracellular activator of integrins to be identified, and it contains an amino-terminal FERM domain (4.1-erythrin–radixin–moiesin domain) complemented with a long rod-like domain at its carboxy-terminal end [3,4]. The later identification of the kindlin family of proteins revealed them to be key co-activators of integrins, alongside talin. Talin and kindlin interact directly with the cytoplasmic tails of integrin β-subunits, with kindlin binding to a membrane-distal NPxY motif and talin binding to a membrane-proximal NPxY motif [5–8]. Biophysical data indicate that talins and kindlins are capable of binding simultaneously to their respective NPxY sites [5], while kindlins may be earlier recruited to integrins during activation than talin is [9].

In humans, three isoforms of kindlin proteins, kindlins-1, -2, and -3, have been identified and shown to adopt tissue-specific patterns of expression. Kindlin-1 is expressed in the epithelial cells of the skin and gut, kindlin-2 is widely expressed but most notably in striated and smooth muscle, and kindlin-3 is expressed particularly in haematopoietic tissues but also in endothelial cells [10,11]. Abnormal expression of, or mutations in, kindlins can result in severe disease. For example, kindlin-1 mutations are associated with Kindler syndrome, a rare genetic dermatitis [12], whereas aberrant expression of kindlin-2 is associated with oncogenesis, especially on relocation to the nucleus where it appears to have a role in the control of transcriptional activity leading to the loss of tumour-suppressor signals [13,14].

In contrast, a nuclear role has not been shown for kindlin-3, but it is overexpressed in chronic lymphocytic leukaemia (which led to its discovery) [15] and when overexpressed in breast cancer, it may play a key angiogenetic role [16]. The importance of kindlin-3 in blood clot formation is, however, clear — for example, mice carrying a QW > AA substitution in the integrin-binding F3 subdomain have compromised clotting ability [17] because kindlin-3 activity is essential for platelet activation [18]. As detailed above, nuclear localisation of kindlin-2 is oncogenic [13,14,19], and another recent intriguing insight is that kindlin-2 is the preferred binding partner of β_1_ integrins over kindlin-3 [20]. This suggests that one possible mechanism linking the two is that overexpression of kindlin-3 could dislodge kindlin-2 from an integrin-bound state, leading to its nuclear relocalisation.

In any case, membrane binding by kindlins is thus not only an important factor in integrin activation, but may also determine their cell signalling impact and even the cell's fate. The membrane-binding capacity of kindlins has been proved to be essential for integrin activation and is dependent on their PH domains [4,21–23] (see Supplementary Figure S1 for a sequence alignment) and on a long loop within the F1 FERM subdomain that is conserved and similar to an equivalent loop in talin [24]. Deletion of the kindlin-3 PH domain eliminates its ability to participate in the adhesion and migration of B cells mediated by the leucocyte integrin LFA-1 [25].

Our previous work on the kindlin PH domains focused on kindlin-1, where we solved its crystal structure to reveal an isoform-specific salt bridge occluding the canonical inositol phosphate-binding site [23]. Molecular dynamics indicated that the salt bridge is dynamic and led us to make a mutant lacking it which had altered inositol phosphate-binding properties. We showed, however, that in either case, the affinity for inositol phosphate ligands is relatively weak (10^−4^ M), although with a specific preference for PtdIns(3,4,5)P_3_. We also showed that the apparent affinity was influenced by the buffer choice for an experiment: phosphate-based buffers seem to interfere with the apparent affinity measured, suggesting a lack of specificity in the PtdIns(3,4,5)P_3_ interactions taking place [23].

Here, we report the crystal structure of the kindlin-3 PH domain together with molecular dynamics-based and biophysical characterisation of its inositol phosphate lipid-binding properties. We show that the kindlin-3 PH domain has a hydrogen bond-based occlusion to its (canonical) PtdInsP-binding cleft, and that, like the kindlin-1 PH domain, it binds surface-immobilised inositol phosphates with rather low (10^−4^ M) affinity. Using lipid nanodiscs as a model membrane system and molecular dynamics simulations to study the interaction of the kindlin-3 PH domain with different combinations of lipid species including inositol phosphate lipids, we show that lipid clustering is likely to be a significant factor in its binding of PtdIns(3,4,5)P_3_ in bilayer membranes. On this basis, we propose that a subset of PH domains is capable of binding to multiple inositol phosphates simultaneously and so via an avidity effect has its interaction with target membranes strengthened. This would explain how an apparently low affinity for inositol phosphates can be biologically relevant to the localisation of kindlins to the plasma membrane and their subsequent activation of integrins.

## Results

### Overall structure of kindlin-3 PH domain

**Figure 1.**
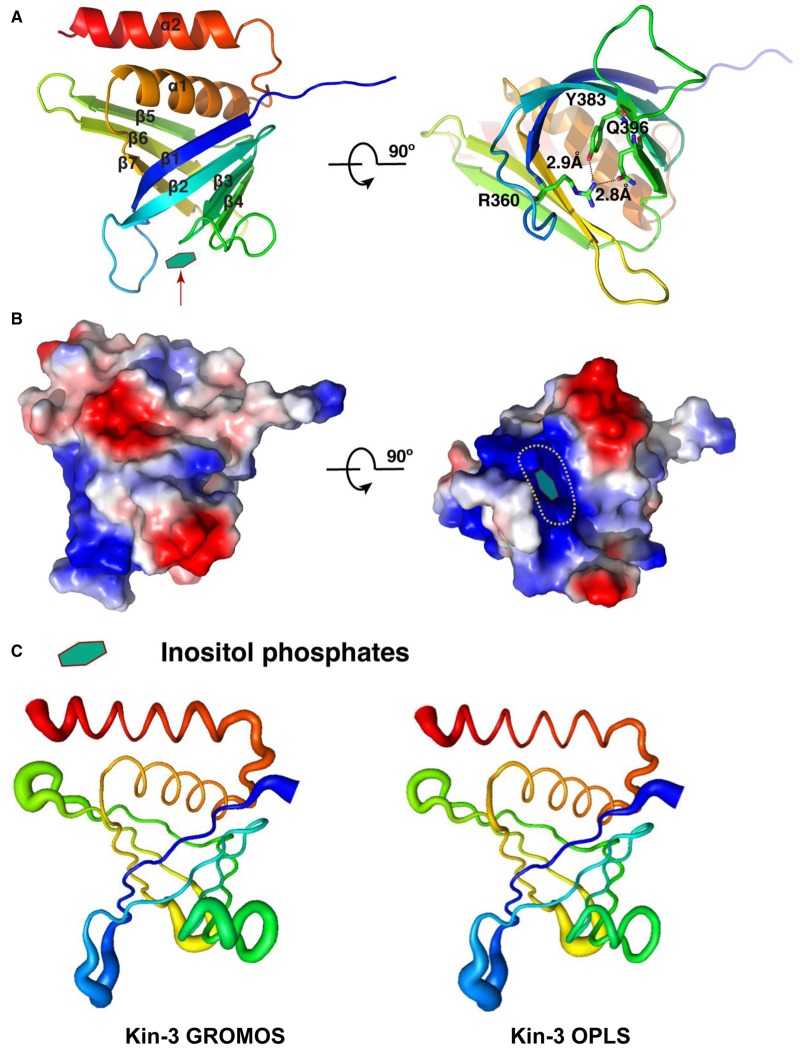
Overall structure of kindlin-3 PH domain. (**A**) Crystal structure of the kindlin-3 PH domain. The protein is coloured rainbow-wise from its amino (blue) to carboxy (red) terminals, and the canonical inositol phosphate-binding site is highlighted with an arrow (left). The hydrogen bonds within the open end of the PH domain are shown (right). (**B**) Surface electrostatic charge of the kindlin-3 PH domain. (**C**) Average root mean square fluctuation (RMSF) of the kindlin-3 PH domain during atomistic MD simulations in water (i.e. in the absence of a bilayer) for two different force fields (i.e. GROMOS and OPLS). The magnitude of the average RMSF of each residue was mapped on the kindlin-3 PH domain crystal structure.

The crystal structure of the kindlin-3 PH domain exhibits an archetypal pleckstrin homology (PH) superfamily fold at its core [26], with a partly open seven-stranded β-barrel capped at one end by a C-terminal α-helix (Figure 1; see Table 1 for data collection and refinement statistics). Similar to that of kindlin-1 and -2 [22,23], the PH domain of kindlin-3 contains an additional C-terminal amphipathic helix extension, which is not present in other PH domains (Figure 1A). A salt bridge in the kindlin-1 PH domain (Arg^380^–Glu^416^) has been shown to occlude its canonical phosphoinositol-binding site [23], and in the kindlin-3 PH domain, this salt bridge is replaced by a hydrogen bond (Arg^360^–Gln^396^) in the equivalent position (Figure 1A). As shown in Figure 1B, the kindlin-3 PH domain has a mixed surface charge distribution with a pronounced concentration of positive charge at the canonical inositol phosphate-binding site.

**Table 1:** Data collection and structure refinement

Data collection
Space group	*P*2_1_2_1_2
Cell dimensions	
*a*, *b*, *c* (Å)	132.04, 36.19, 52.69
*α*, *β*, *γ* (°)	90, 90, 90
Resolution (Å)	31.73–2.23 (2.31–2.23)
*R*_merge_	0.049 (0.432)
*I*/*σI*	23.7 (2.3)
Completeness (%)	96 (93)
Redundancy	3.1 (2.6)
CC_half	0.998 (0.771)
Refinement	
Resolution (Å)	31.73–2.23 (2.31–2.23)
No. of reflections	38 032 (3014)
No. of unique reflections	12 441 (1155)
*R*_work_/*R*_free_	21.94 (27.07)
No. of atoms	
Protein	2123
Ligand/ion	1
Water	13
B-factors (Å^2^)	
Protein	84.92
Ligand/ion	13.06
Water	61.66
R.m.s. deviations	
Bond lengths (Å)	0.007
Bond angles (°)	1.11
Ramachandran	
Favoured (outliers)	98% (0%)

The overall fold of the kindlin PH domain appears remarkably conserved. The kindlin-3 PH domain is closest in structure to the PH domain of kindlin-2 (RMSD = 0.69 Å), with the major conformational difference being in the β1–β2 loop. The close relationship between the kindlin PH domain structures is reflected in their position within a structural phylogenetic tree we constructed for a set of PH domains of different kinds of function and (where relevant) with different lipid-binding properties (Supplementary Figure S2). Of note, a previously constructed structural phylogeny places the previously determined kindlin-3 PH domain NMR structure in a different relationship to that of kindlins-1 and -2 to the crystal structure reported here [23]. This highlights the sensitivity of structural phylogenetic comparisons to the quality of the models used.

PH domains with canonical inositol phosphate-binding sites typically exhibit a highly positively charged core at the open end of the PH domain, whereby they interact with their ligand, as seen in PH domains of Grp1 [27] and PLCδ1 [28]. Non-canonical PH domains, such as Slm1 [29], Tiam1, and ArhGAP9 [30], lack the same kind of positive charge distribution and instead have their inositol phosphate-binding site on the side of the barrel mainly between the β1–β2 loop and the β5–β6 loop (Supplementary Figure S3). The PH domains of kindlins-3 and -2 have similar surface charge distribution to the PH domains with canonical inositol phosphate-binding sites (Figure 1B), indicating that inositol phosphate might interact in a canonical way through the β1–β2 loop. The previously reported crystal structure of the kindlin-1 PH domain is not as complete as either of the available crystallographic kindlin-2 or -3 models, lacking seven residues within the β1–β2 loop; its surface charge characteristics, however, seem to be similar to those of the other kindlin PH domains.

Atomistic molecular dynamics (AT-MD) simulation of the kindlin-3 PH crystal structure in solution (i.e. in the absence of any lipid bilayer) with two different force fields showed that the overall fold of the PH domain is retained during the simulations. The seven-stranded β-barrel in the core of the PH domain is very stable, whereas the unstructured regions connecting the β-strands are somewhat more flexible (Figure 1C). In particular, the β1–β2 loop is flexible with both simulation force fields, in good agreement with the crystal structure (Figure 1A). At the end of the simulation, the side chains of the positive residues on the β1–β2 loop remain exposed towards the open end of the barrel, presumably providing a site for inositol phosphate binding. With the exception of one simulation in which the Arg^360^–Gln^396^ hydrogen bond identified in the crystal structure breaks for an extended period of time, the Arg^360^–Gln^396^ hydrogen bond was generally preserved during the simulations although it breaks transiently before re-forming (Supplementary Figure S4).

### Molecular dynamics simulations reveal a membrane-bound state of kindlin-3 PH domain and its interaction with PIP lipids

Coarse-grained molecular dynamics (CG-MD) simulations can be used to identify the molecular mechanism of peripheral membrane proteins binding to model membranes incorporated with anionic, for example, PIP lipids [31,32]. In particular, MD simulations have been used to study the interaction of many PH domains, e.g. DAPP1 PH [32] and Grp1 PH [33], with PIP lipids at the molecular level. A recent study has also demonstrated that MD simulations can be used to study the free energy of the interaction of the Grp1 PH domain with PIP_2_ and PIP_3_ molecules [34].

**Figure 2.**
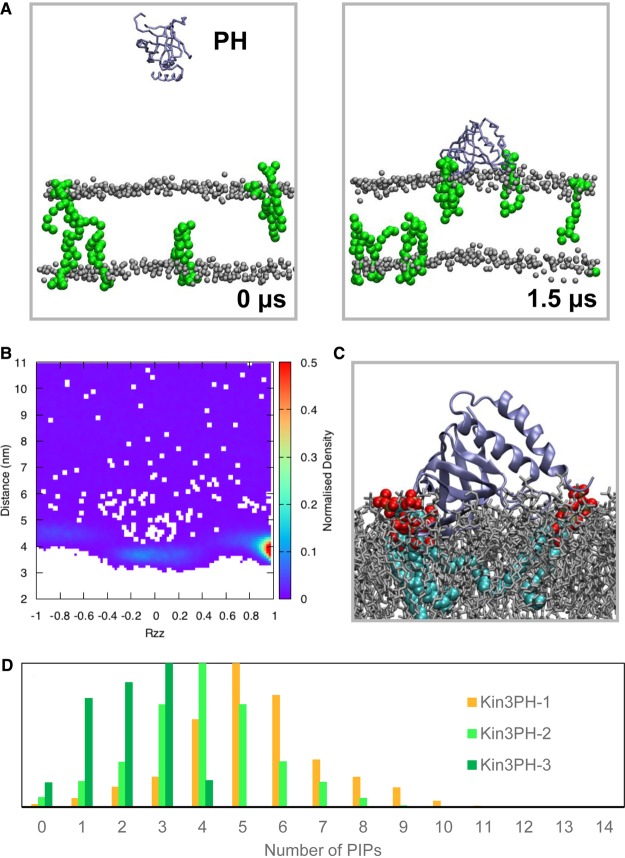
Coarse-grained MD simulations of the kindlin-3 PH domain interacting with a PIP-containing lipid bilayer. (**A**) Snapshots at the beginning and at the end of one of the simulations with 4 PIP_3_ molecules in each leaflet (Kin3PH-3 in Table 2). PIP_3_ molecules are shown in green, POPC and POPS phosphate atoms are shown in grey, and the kindlin-3 PH domain is shown in ice blue. (**B**) Rotation of the kindlin-3 PH domain (shown as the R_zz_ component of its rotational matrix) relative to the distance between the centre of mass of the protein and the centre of mass of the bilayer. (**C**) Final snapshot from one of the atomistic simulations that started using snapshots from the simulations shown in (**A**). (**D**) Number of PIP molecules interacting with the kindlin-3 PH domain. The number of PIP lipids is shown for Kin3PH-1, Kin3PH-2, and Kin3PH-3 simulations (Table 2) in orange, green, and light green, respectively. For this calculation, a PIP molecule is considered to be in contact with the protein if its phosphate is within 1 nm of the protein. The number of PIP molecules is shown only for the frames in which the protein is bound to the bilayer (i.e. the *z* component of the distance between the protein centre of mass and the bilayer centre of mass is smaller than 4.75 nm).

To examine the association of the new crystal structure of the kindlin-3 PH domain with model membranes, the PH domain was displaced away from a preformed bilayer containing 4 PIP_3_ molecules in each leaflet (concentration of ∼1.5% that mimics the *in vivo* concentration of PIP_3_ lipids in the plasma membrane), and an ensemble of 20 CG-MD simulations of 1.5 µs each was performed (Figure 2A). Calculation of the orientation of the PH domain relative to the bilayer (by calculating the R_zz_ component of its rotational matrix) suggests that the PH domain has a preferred orientation relative to the bilayer (Figure 2B). In this orientation, residues 360–372 (i.e. the positively charged loop between strands β1/β2) made the largest number of contacts with the PIP_3_ molecules. Regions 385–386, 410–415, and 434–436 also face towards the bilayer (Figure 2) and interact with PIP_3_ molecules. This orientation is rather different from, for example, the canonical orientation of the Grp1 PH domain relative to a bilayer [27,35]. Indeed, the helix at the end of the Grp1 PH domain was shown to be in a parallel orientation relative to the bilayer. We note, however, that in our ensemble we also observe secondary binding modes in which the kindlin-3 PH domain adopts an orientation that is similar to that of the Grp1 PH domain (Supplementary Figure S5E). Atomistic simulations starting from the preferred orientation of the PH domain on the PIP_3_-containing bilayer reveal that the PH domain retains this orientation relative to the bilayer.

**Table 2:** Summary of simulations

Simulation	Bilayer composition	Duration
Coarse-grained		
Kin3PH-1	POPC:POPS:PIP_2_ (∼73:20:7)	20 × 1.5 µs
Kin3PH-2	POPC:POPS:PIP_3_ (∼73:20:7)	20 × 1.5 µs
Kin3PH-3	POPC:POPS (∼78:20) + 4 PIP_3_ molecule per leaflet	20 × 1.5 µs
Kin3PH-4	POPC:POPS (∼79:20) + 2 PIP_3_ molecule per leaflet	20 × 1.5 µs
Kin3PH-5	POPC:POPS (∼79:20) + 1 PIP_3_ molecule per leaflet	20 × 1.5 µs
Kin3PH-6	POPC:POPS (∼73:20) + 5% PIP_2_ and 4 PIP_3_ molecule per leaflet	20 × 1.5 µs
Kit3mutPH-1	POPC:POPS (∼78:25) + 4 PIP_3_ molecule per leaflet — K363A, K367A mutation	20 × 1.5 µs
Atomistic		
Kin3PH_AT-1	No bilayer — GROMOS force field	3 × 100 ns
Kin3PH_AT-1	No bilayer — OPLS force field	3 × 100 ns
Kin3PH_AT-3	POPC:POPS:PIP_2_ (∼73:20:7)	6 × 100 ns
Kin3PH_AT-4	POPC:POPS (78:20) + 4 PIP_3_ molecule per leaflet	4 × 100 ns

To examine whether the concentration of PIP_3_ in a membrane bilayer affects the association of the kindlin-3 PH domain to the membrane, MD simulations were then carried out with various concentrations of PIP_3_ lipids in the bilayer (7% PIP_3_ or 2% PIP_3_ in each leaflet, or 1% PIP_3_ in each leaflet; see Table 2). Despite the fact that when we reduce the number of PIP_3_ molecules in the bilayer, the kindlin-3 PH domain associates with the bilayer less frequently, in all cases it adopts a preferred orientation which is similar to the preferred orientation described above (Supplementary Figure S6). The PH domain adopted the same orientation when we ran simulations with 7% PIP_2_ lipids or with both PIP_3_ and PIP_2_ lipids in the bilayer (Kin3PH-1 and Kin3PH-6 in Table 2, respectively). Interestingly, atomistic simulations starting from four different snapshots of the kindlin-3 PH domain bound to PIP_2_-containing bilayers at the preferred orientation (six simulations overall; see Materials and Methods) resulted in three simulations in which the kindlin-3 PH domain retained its orientation relative to the bilayer and in three simulations in which the PH domain adopted the secondary binding mode at the end of the atomistic simulations (Supplementary Figure S5) as in subsequent potential of mean force (potential of mean force, PMF) calculations (see below).

Analysis of the contacts between the PIP lipids and the PH domain during the simulations suggested that residues R362, K363, and K367 and residues K363, K367, and R370 made the largest number of contacts with PIP_2_ and PIP_3_ lipids, respectively. Our analysis also revealed that the PH domain induces clustering of the PIP lipids when it binds to the bilayer (Figure 2D). The clustering of PIP_2_ lipids is somewhat higher compared with PIP_3_ lipids. Note that the clustering of the PIP lipids occurs only in the leaflet in which the protein is bound. In the simulations with 7% of PIP_2_ or 7% PIP_3_ lipids in the bilayer, most of the time we observe four PIP_3_ and five PIP_2_ lipids, respectively, in an annulus of 1 nm from the protein surface. Given that recent studies have suggested an important role for anionic lipids and PIPs in integrin activation [36,37], the change in the local lipid environment by the binding of the PH domain may have some functional role.

### Free energy of binding of the kindlin-3 PH domain to PIP(4,5)P_2_ and PIP(3,4,5)P_3_

**Figure 3.**
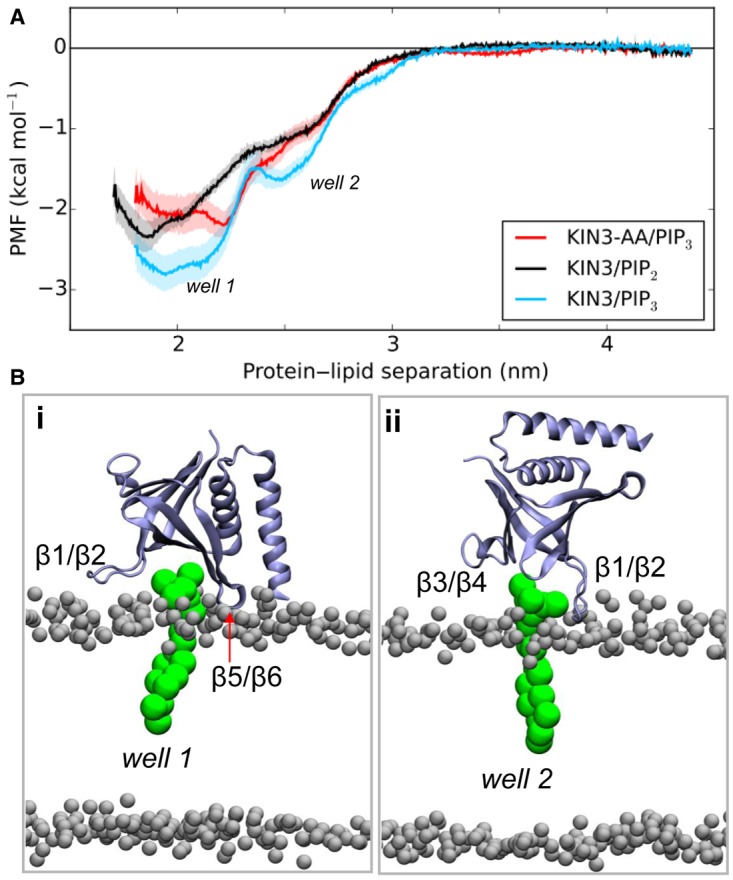
PMF calculations of kindlin-3 PH domain with PIPs. (**A**) PMF calculations for the binding of the WT and the mutated form of the kindlin-3 PH domain to PIP_2_ (black) and PIP_3_ (cyan and red) molecules. (**B**) Kindlin-3 PH/bilayer complexes corresponding to wells 1 and 2 in (**A**). The PIP_3_ molecules are shown in green and the PH domain in ice blue. For this calculation, the initial structures of WT or mutant kindlin-3 PH domains bound to PIP_3_ or PIP_2_ in a 80:20 POPC:POPS bilayer were generated by alignment with an existing Grp1–PH/PIP_3_ complex. The PH domain was pulled from the bound PIP lipid and bilayer along the membrane normal (*z*-axis). See Materials and Methods for more details.

Our analysis above revealed the molecular mechanism of the association of the kindlin-3 PH domain with the membrane, suggesting the occurrence of two different modes of interaction of the PH domain with the PIP-containing bilayer. To examine the strength of interaction of the PH domain with PIP_2_ and PIP_3_ molecules, we performed PMF calculations using a protocol we developed recently (see Materials and Methods and ref. [34]). PMF profiles for PIP_3_ and PIP_2_ lipids have a global minimum with a well depth of approximately −3 kcal/mol for PIP_3_ and a well depth of approximately −2.5 kcal/mol for PIP_2_ lipids. This suggests that the protein associates somewhat more strongly with PIP_3_ lipids (Figure 3). The first well (corresponding to the preferred orientation of the PH domain) in the profile of the PIP_3_ molecules was followed by a second shallower well (corresponding to a secondary PH orientation) at a distance of ∼2.5 nm. Examination of the interactions of the kindlin-3 PH domain with the lipids showed that, in the windows covering ∼1.5 to ∼2.3 nm protein–lipid separations, the protein is in a similar orientation to the preferred orientation (mode 1) described above in which the protein interacts with the bilayer mainly via the β1/β2 unstructured loop. In these windows, the PIP_3_ molecule is located between the β1/β2 and β5/β6 loops (i.e. in a non-canonical PIP-binding site) for most of the time (Supplementary Figure S7A). We note that we have also seen interactions of PIP molecules with this site in our encounter simulations as described above. Interestingly, in the second well the protein adopts an orientation that is similar to the secondary binding mode also observed in the simulations above, with the PIP_3_ molecule now located between the β1/β2 and β3/β4 loops (i.e. in the canonical PIP-binding site). In contrast, the profiles for PIP_2_ lipids and for the mutated form of kindlin-3 PH domain did not have a second well. In the simulations with a PIP_2_ molecule, the PIP_2_ is found both in the canonical (i.e. contacting the β1/β2 and β3/β4 loops) and in a non-canonical site (i.e. contacting the β1/β2 and β5/β6 loops; Supplementary Figure S7B). We note that the depth of the well for the binding of the Grp1 PH domain to PIP lipids using the same method was −5.3 kcal/mol for PIP_3_ and −3.8 kcal/mol for PIP_2_, suggesting that the binding of the kindlin-3 PH domain is weaker compared with the Grp1 PH domain binding a single lipid [34]. Disruption of the PH/PIP interactions by mutating the K363 and K367 residues which were identified to form a large number of contacts with the PIP lipids resulted in a reduced binding to PIP_3_ molecules by −1 kcal/mol.

### An SPR study confirms that the kindlin-3 PH domain binds to multiple phosphatidylinositol phosphates

**Figure 4.**
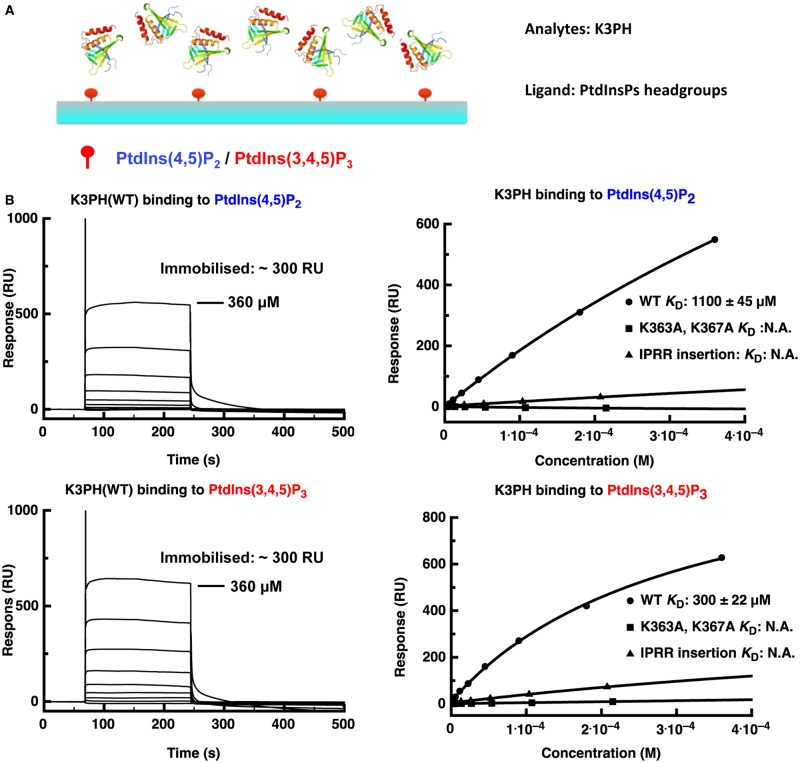
SPR study of the kindlin-3 PH domain with immobilised PIP2 and PIP3 headgroups. (**A**) Schematic representation of SPR experiments with the immobilised lipid headgroup as the ligand and the PH domain as the analyte. (**B**) SPR measurements of the kindlin-3 PH domain WT and mutants with immobilised PtdIns(4,5)P_2_ and PtdIns(3,4,5)P_3_, respectively. On the left in each case, we show the background-subtracted raw sensogram data for the WT protein, while on the right we show binding saturation curves for WT, K363A K367A and IPRR insertion mutants. The highest concentration used is given on each set of sensogram curves; subsequent binding profiles derive from a series of twofold dilutions.

To verify the potential interaction between clustered phosphatidylinositol phosphates and the kindlin-3 PH domain observed in our MD simulations, we conducted surface plasmon resonance (SPR) experiments. Firstly, a biotinylated inositol phosphate was immobilised onto a streptavidin-derivatised sensor chip, as in previous studies [23]. The use of streptavidin-mediated immobilisation meant that the distance separating individual lipids would be larger than the size of the tetrameric streptavidin, and therefore than the PH domain itself, meaning that one PH domain would be able to bind no more than one lipid headgroup simultaneously, imposing one-to-one binding in the interaction (Figure 4A). In agreement with the MD simulation results, the kindlin-3 PH domain displays a preference for PIP_3_, though with a dissociation constant (*K*_D_) of ∼300 µM, in agreement with previous measurements [23]. The apparent binding capacity for PIP_2_ was much lower (*K*_D_ = 1100 µM). Introduction of two point mutations (K363A and K367A) effectively abolished the binding capacity of both inositol phosphate moieties, indicating that the interaction is specific even if it is low affinity (Figure 4B). We note that, in the MD simulation with the mutated form of the kindlin-3 PH domain, the protein is capable of binding to the bilayer in a similar orientation to the wild type (WT); however, in many simulations, it quickly dissociates from the bilayer (Supplementary Figure S8). A naturally occurred IPRR (isoleucine–proline–arginine–arginine) insertion in the β1–β2 loop of kindlin-3 has been reported to disable integrin activation and thus lymphocyte adhesion and migration [21]. In agreement with results obtained by others using a pull-down assay with IP_3_ and IP_4_-coated beads, our SPR study *in vitro* revealed that the IPRR insertion directly disrupted the interaction between the PH domain and phosphoinositol moieties (Figure 4B,C), presumably because of the steric effect of an elongated β1–β2 loop.

The observation in MD simulation that multiple phosphoinositol molecules may bind to more than one site on a single PH domain (Figures 2 and 3) led us to attempt further verification of this mode of interaction experimentally. Nanodiscs have been shown to be an effective model membrane system due to their stability and capacity to mimic the membrane bilayer [38,39], including the fluidity of lipids though within a limited bilayer area. To study the effect of phosphatidylinositol lipid concentrations on their binding by PH domains, we performed the SPR experiments in an inverted set-up. The biotinylated PH domain was immobilised onto the sensor chip and the nanodiscs were prepared as the analyte.

Nanodiscs incorporated with different components were stable in solution (Supplementary Figures S8D and S9A), with an estimated molecular mass of 120 kDa; they behaved as single species with a sedimentation coefficient of 4 S as assessed by analytical ultracentrifugation (Supplementary Figure S9B). It appears that the diameter of the nanodiscs correlates with the molar ratio of phosphatidylinositides incorporated in their membranes: nanodiscs with 5% PtdIns(3,4,5)P_3_ (diameter, *d* = 5.87 nm) were bigger than those with 3% PtdIns(3,4,5)P_3_ (*d* = 4.64 nm; Supplementary Figure S9C,E). Note that the diameters are small because they are average values; as shown in Supplementary Figure S9D, the nanodiscs are elliptical and of the expected size.

**Figure 5.**
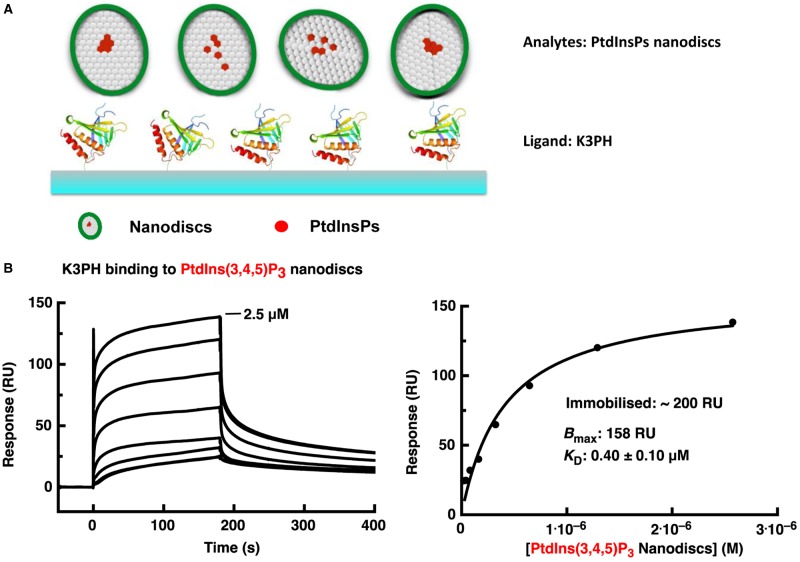
SPR study of the kindlin-3 PH domain with PtdIns(3,4,5)P_3_ incorporated nanodiscs. (**A**) Schematic representation of SPR experiments with the immobilised PH domain as the ligand and lipid nanodiscs as the analyte in solution. (**B**) SPR measurement between the WT kindlin-3 PH domain and nanodiscs with 5% PtdIns(3,4,5)P_3_. The background-subtracted raw sensogram data for the WT protein are shown on the left and the resulting binding saturation curve on the right. The highest concentration used is given on the sensogram graph; subsequent binding profiles derive from a series of twofold dilutions. See Materials and Methods for experimental details.

The SPR results showed that 5% PIP_3_-incoporated nanodiscs bind to the kindlin-3 PH domain strongly, with a much-enhanced affinity (*K*_D_ = 0.4 µM; Figure 5) compared with that shown by the PH domain towards immobilised inositol phosphates. In contrast, nanodiscs with 5% PIP_2_ showed no binding to the kindlin-3 PH domain, demonstrating that PIP_3_ is its real ligand and suggesting that the apparent binding to immobilised PIP_2_ is due to a non-specific interaction. We also performed the same experiments with nanodiscs without incorporated phosphatidylinositol moieties and observed no binding in SPR experiments.

There is evidence for PIP_2_ clustering within lipid bilayers [40], while other data suggest protein-driven PIP_2_ clustering in signalling complex formation [41]. Furthermore, while a study of yeast proteins has revealed that the interaction between one set of PH domains and membranes can be enhanced by co-operative interaction between PIPs and other lipids (such as POPS) [42], the interaction between the kindlin-3 PH domain and PIP_3_ seems to more rely on the concentration of PIP_3_ in the membrane, i.e. co-operativity among copies of the same lipid.

### Grp1 PH domain binds inositol phosphate with 1:1 specificity

**Figure 6.**
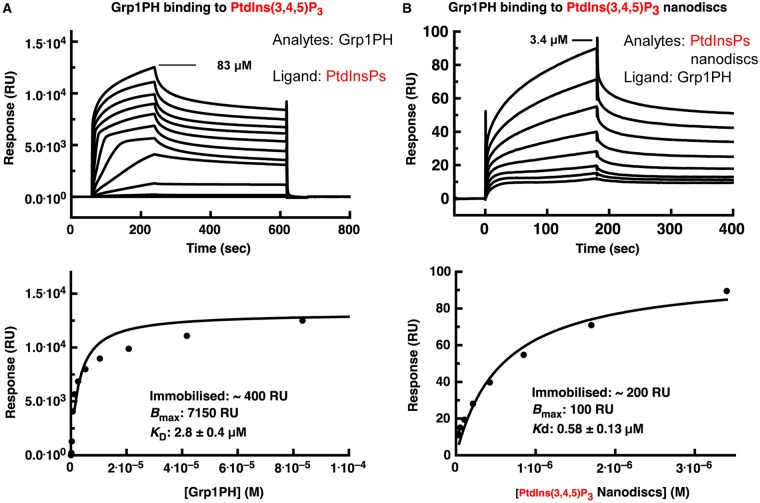
SPR study of interaction between the Grp1PH domain and PtdIns(3,4,5)P_3_ in two experimental set-ups. (**A**) Immobilised PtdIns(3,4,5)P_3_ headgroup as the ligand and the PH domain as the analyte. (**B**) Immobilised Grp1PH domain as the ligand and lipid nanodiscs as the analyte. The upper panel in each case gives the background-subtracted raw sensogram data and the lower panel gives the resulting binding saturation curve. The highest concentration used is given on the sensogram graphs (83 µM Grp1 PH domain in **A**; 3.4 µM nanodiscs in **B**); subsequent binding profiles derive from a series of twofold dilutions. See Materials and Methods for experimental details.

We further asked whether our observations are specific to the kindlin-3 PH domain (and similar domains) or could also be shown for PH domains which are already known to bind to inositol phosphate species in a classical, 1:1 interaction mode. If they could be shown for such well-characterised domains, then our results with the kindlin-3 PH domain would seem questionable. The crystal structure of the Grp1 PH domain in complex with Ins(1,3,4,5)P_4_ [equivalent to the PtdIns(3,4,5)P_3_ headgroup] reveals the ligand well embedded in a pocket formed by positively charged residues [43]. In line with this, we showed by SPR that the analyte/ligand design of our experiment does not significantly affect the apparent affinity of Grp1 for PIP_3_ compared with the effect it has on the kindlin-3 PIP_3_ binding observed. The Grp1 PH domain was shown to bind to immobilised PIP_3_ headgroups with a *K*_D_ of 2.8 ± 0.4 µM (Figure 6A), and to immobilised PIP_2_ headgroups with a *K*_D_ 10 times greater (Supplementary Figure S10), consistent with a previous study though with a lower absolute value due to methodological differences [27,44]. In contrast, the binding affinity to PIP_3_ within nanodiscs (5% of constituent lipids) has a *K*_D_ of 0.58 µM. So, in the case of Grp1, there is only a five times difference in affinity between immobilised lipid headgroups and whole lipids in a lipid nanodisc (rather than the ∼100× enhancement observed with the kindlin-3 PH domain). This suggests that the Grp1–PH interaction with PIP_3_ at high affinity does not rely on the binding of multiple PIP_3_, though it does indicate an enhancement of lipid binding — most probably by a simple electrostatic attraction — in a membrane-like context [42].

## Discussion

The cytoplasmic surface of the plasma membrane serves as a platform for many critical biological processes. The recruitment of peripheral membrane proteins from the cytoplasm to the plasma membrane surface is spatiotemporally and accurately regulated. The PH domain is one of a selection of commonly used folds directing protein–membrane association and has been shown to confer specificity to different inositol phosphates on protein–membrane interactions.

PH domains have been shown to bind phosphatidylinositol phosphates (PtdInsPs) with different degrees of specificity [45] and the PH domains of the kindlins have a limited affinity for them [23], although the membrane interaction mediated by the PH domain is essential for integrin activation [23]. Phosphatidylinositol lipids are present in membranes at different concentrations, which may lead to variable activity and roles in membrane structure and membrane recognition. PtdIns(4,5)P_2_ (PIP_2_) has been reported to be the most abundant phosphatidylinositol lipid in cells [46] and can undergo phosphorylation to a PtdIns(3,4,5)P_3_ (PIP_3_) state; both PIP_2_ and PIP_3_ are found at the plasma membrane. Monophosphorylated inositide lipids are, in contrast, associated with compartments such as the Golgi and endoplasmic reticulum [46].

In the present study, we firstly solved the crystal structure of the kindlin-3 PH domain and then used the crystal structure as the basis for a multiscale molecular dynamics simulation study. MD simulation revealed that kindlin-3 PH domain interacts with the PIP lipids primarily via the loop connecting the β1 and β2 strands, and that it has a somewhat higher affinity for binding PtdIns(3,4,5)P_3_ than for PtdIns(4,5)P_2_ in a membrane-like system. Moreover, one kindlin-3 PH domain appears to bind multiple inositol phosphate lipids, at both canonical and non-canonical sites, by means of which the affinity of interaction between the PH domain and the membrane is greatly enhanced by an avidity effect. In contrast, with a PH domain (from Grp1) previously shown to have canonical and specific one-to-one binding to inositol phosphate lipid, this one-to-more binding mode was not observed. These observations from MD simulation were supported by our SPR experiments in two complementary set-ups. The first one involves immobilisation of biotinylated inositol phosphates on the sensor chip and use of the PH domain (WT and mutant) as the analyte, to mimic an isolated one-to-one binding mode. In this case, each PH domain could only bind to no more than one phospholipid due to the distance restraints imposed by streptavidin-mediated immobilisation. The other set-up attempted to mimic the bilayer plasma membrane, by immobilising the PH domain on the chip and using soluble membranes (nanodiscs) as the analyte. The affinity of interaction measured between the PH domain and inositol phosphate lipids was remarkably affected by experimental design.

Compared with liposomes, nanodiscs have obvious advantages for protein–lipid interaction studies in a membrane-like environment: they are more stable and have a limited number of lipids per disc. Nanodiscs made of MSP1E3D1 are estimated to accommodate ∼125 POPC (1-palmitoyl-2-oleoyl-*sn*-glycero-3-phosphocholine) lipids, with a diameter of ∼13 nm [47]; this is in agreement with our cryo-EM map of the nanodisc (Supplementary Figure S9D), which is fitted well (length ∼14 nm and width ∼9 nm) with the elongated and twisted conformation of the human apolipoprotein A1 [48]. In the SPR experiments here, the small and homogeneous size of the nanodisc membrane is critical for 1:1 model fitting and interpretation of the SPR results. Liposomes, with inherently larger diameters (e.g. 50–100 nm), would create multiple possible contact sites for PH domains, confounding binding affinity analysis. Nanodiscs, however, are capable of binding no more than one immobilised PH domain due to their limited size compared with the size and density of immobilised PH domains on the chip surface.

We find that PtsIns(3,4,5)P_3_ clustering enhances the affinity of kindlin PH domains for target membranes. This is a charge-based interaction as shown by its loss when positively charged lysine residues are mutated to alanine (Figure 4). Similarly, many other proteins have been shown to interact with clusters of PtdInsP_2_ lipids on the basis of opposite charge attraction. For example, a series of basic residues in the MARCKS peptide from a protein kinase C substrate are known to bind to PtdIns(4,5)P_2_ lipid clusters, as do syntaxin-1A, BAR domains, and N-WASP. N-WASP is an activator of Arp2/3, which in turn nucleates actin filament formation at membranes [49,50].

There is increasing evidence showing that PH domains can interact with multiple lipid species simultaneously. A recent crystal structure of the ASAP1 PH domain suggested that there were two anionic lipid-binding sites on the ASAP1 PH domain (a canonical site and an atypical site) [51], and recent comparative MD simulations have suggested that both sites are present for many PH domains [33]. Moreover, Vonkova et al. [42] studied 91 different PH domains and showed that lipid co-operativity is essential for their recruitment to the membrane. Clustering of lipids by the kindlin-3 PH domain is expected to change the local lipid environment. Given that kindlins act in synergy with talin, which was also shown to interact with anionic lipids [52], this may provide a mechanism for how talin and kindlins, in turn, associate with the integrin. Focal adhesion kinase has also been shown to bind to inositol phosphates, inducing clustering of a protein-mediated kind [2,23].

**Figure 7.**
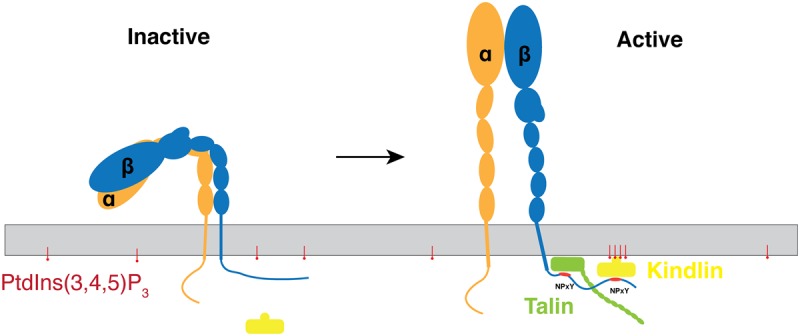
A proposed model for integrin activation by kindlin-3 with recruitment by clustered inositol phosphates to a membrane. The activating conversion of integrins to an upright conformation is promoted by the binding of talin to the membrane-proximal NPxY and of kindlin to the membrane-distal NPxY on an integrin β-subunit cytoplasmic tail. Clustered PtdIns(3,4,5)P_3_ interacts with and recruits kindlin-3 from the cytosol to the membrane, thus facilitating the formation of an integrin–talin–kindlin ternary complex.

The orientation of the kindlin-3 PH domain on a target membrane indicated by this study, and the fact that kindlins have a rather long unstructured loop in the F1 domain allow us to form a hypothesis for how the kindlin head domain may interact with the membrane and with the integrin receptor. In particular, the orientation of the PH domain that we find, in combination with the short linker regions plugging it into the F2 subdomain, suggests that it acts as a buffer or spacer between the kindlin and the membrane surface, and thereby allows the F3 subdomain to interact with the membrane-distal NPxY integrin β-subunit tail motif rather than the membrane-proximal NPxY bound by talin [53] (Figure 7). Thus, in combination with the N-terminal F0 domain [54] and the long F1 loop [24], the PH domain provides kindlins with a specific location and binding mode for integrin activation.

There are no high-resolution structural data for the rest of kindlin-3; however, it has been shown to have a similar structure to the talin head domain [5]. Therefore, further studies will focus on structure determination for kindlins and use structural and biophysical methods to test the hypothesis outlined above that binding of clustered phosphatidylinositol phosphate lipids is a mechanism employed by some proteins and specifically a subset of PH domains to enable the binding of membranes with an enhanced affinity.

## Materials and methods

### Constructs design, cloning, and mutagenesis

Mouse *FERMT3* (UniProtKB: Q8K1B8) encoding kindlin-3 PH domain (residues 344–479) and Grp1 (general receptor of phosphoinositides 1, UniProtKB: O43739) PH domain (residues 264–392) were cloned into a pOPINJ vector, which contains a tandem His_6_ and GST (glutathione *S*-transferase) tag followed by a Human Rhinovirus-3C protease cleavage site in the N-terminus. The successful cloning was confirmed by DNA sequencing (Source Bioscience, U.K.). Mutagenesis and insertions were achieved by overlapping PCR following the standard protocol [55]. A DNA fragment encoding a 15-residue AviTag™ was inserted between the 3C protease cleavage site and the PH domain by PCR to enable *in vivo* protein biotinylation [56] using a BirA+ *Escherichia coli* strain for SPR experiments.

### Expression of kindlin-3 PH domain and Grp1 PH domain

The construct encoding recombinant kindlin-3 PH domain was transformed into *E. coli* B834 strain. A single colony was picked from fresh transformants on an LB-agar–carbenicillin plate and cultured in 100 ml of LB supplemented with 50 µg/ml carbenicillin overnight. The pre-culture was diluted 50 times into LB media with antibiotics and incubated at 37°C with vigorous shaking at 200 rpm until OD_600_ reached ∼0.6. The expression of protein was induced by 1 mM IPTG (isopropyl β-d-1-thiogalactopyranoside) at 18°C overnight. Cells were harvested by centrifugation at 6000 ***g*** for 20 min and pellets were frozen in a −80°C freezer until further use. Other proteins used in the present study were expressed using the same protocol.

### Protein purification and crystallisation

The frozen cell pellet of the kindlin-3 PH domain (∼10 g of cell pellet from 2 l of overnight culture) was resuspended in lysis buffer [50 mM Tris (pH 7.5), 500 mM NaCl, and 0.1% Tween-20] and lysed with sonication, and the debris was separated by centrifugation at 40 000 ***g*** for 1 h. The resulting supernatant was incubated with glutathione sepharose equilibrated with lysis buffer for 2 h. The glutathione sepharose with protein bound was then washed with lysis buffer extensively. GST-3C protease was added to cleave the kindlin-3 PH domain from sepharose overnight at 4°C with gentle shaking. Supernatant containing a PH domain was concentrated and applied to size-exclusion chromatography (Superdex 75 16/60, GE Healthcare Life Sciences) in 10 mM Tris–HCl (pH 7.5), 150 mM NaCl, and 0.7 mM TCEP [*tris*(2-carboxyethyl)phosphine] buffer to obtain the purified proteins. The purification of kindlin-3 PH domain mutants and the Grp1 PH domain and biotinylated proteins was carried out in the same way. Successful *in vivo* biotinylation was verified by efficient binding to streptavidin magnetic beads (Sigma-49532).

Crystallisation screening was performed by means of sitting-drop vapour diffusion methods. Briefly, 100 nl of proteins at 20 mg/ml in 10 mM Tris–HCl (pH 7.5) and 150 mM NaCl were mixed with 100 nl of reservoirs and equilibrated against 95 µl of crystallisation reservoir (Hampton Research) at 293 K. Crystals appeared within 4 days and continued growing until 2 weeks in 4 M sodium nitrate and 0.1 M sodium acetate (pH 4.6). Crystals were flash-cooled with 25% (v/v) glycerol in the mother liquor as a cryoprotectant.

### Data collection, processing, and structure determination

Diffraction data were collected using beamline I24 at Diamond Light Source (Didcot, U.K.) and then indexed, integrated, and scaled using the Xia2 pipeline [57]. The structure of the kindlin-3 PH domain was solved by molecular replacement (MR) using PHASER [58] with a polyalanine model of the kindlin-1 PH domain (PDB: 4BBK). The resulting model from MR was further built by *Buccanneer* [59] and corrected manually in *Coot* [60]. Refinement was accomplished using *PHENIX.refine* together with manual correction in *Coot*. Molecular graphics were prepared with *PyMOL*. Attempts at co-crystallising the kindlin-3 PH domain with Ins(1,3,4,5)P_4_ and at soaking the crystal with highly concentrated Ins(1,3,4,5)P_4_ resulted in a PH domain structure without ligand bound, presumably due to the low-affinity interaction between isolated Ins(1,3,4,5)P_4_ and the kindlin-3 PH domain.

### Nanodisc preparation

POPC and POPE (1-palmitoyl-2-oleoyl-*sn*-glycero-3-phosphoethanolamine) were purchased from Avanti Polar Lipids, and PtdIns(4,5)P_2_ (P-4516), PtdIns(3,4,5)P_3_ (P-3924), and Ins(1,3,4,5)P_4_ (Q-1345) were purchased from Echelon Biosciences. Nanodiscs were prepared following standard protocols [38]. Briefly, to prepare nandiscs containing 5% (mol/mol) PtdIns(3,4,5)P_3_, a mixture of 200 µl of 10 mg/ml POPC, 200 µl of 10 mg/ml POPE together with 670 µl of 0.5 mg/ml PtdIns(3,4,5)P_3_ in chloroform were dried on a clean Pyrex tube under argon. Chloroform residue was then removed by desiccation in a desiccator attached on a VARIO-SP diaphragm pump (Vacuubrand). The lipid film was subsequently hydrated by adding 1 ml of solubilisation buffer [20 mM HEPES (pH 7.5), 150 mM NaCl, and 17 mM Na-cholate] followed by vigorous votexing. The hydrated lipid film was then sonicated for 10 min in a water bath sonicator before 250 µl of MSP1E1 (7 mg/ml) proteins were added to the lipid mixture. Nanodiscs were self-assembled upon removal of Na-cholate by dialysing against 20 mM HEPES (pH 7.5) and 150 mM NaCl buffer overnight. The aggregate was removed by size-exclusion chromatography (Superdex 200 10/300, GE Healthcare Life Sciences), and the peak fractions corresponding to the size of monomeric nanodiscs species were collected (Supplementary Figure S9A) and used immediately for biophysical characterisation and SPR experiments.

### Dynamic light scattering

Dynamic light scattering measurements were conducted on a Protein Solutions DynaPro instrument at 20°C. The nanodisc eluates from size-exclusion chromatography were diluted to ∼0.05 mg/ml in 10 mM HEPES (pH 7.5) and 150 mM NaCl before measurement in a cuvette.

### Cryo-electron microscopy

After plunge-freezing in liquid ethane, nanodiscs on holey carbon grids were imaged using an FEI Tecnai F30 microscope operating at 200 kV and at a nominal magnification of 59 000×. Images were picked using *Boxer* [61] and reconstructed *ab initio* using *IMAGIC* [62] followed by the *SPIDER* [63] software. The resolution of the map was determined by Fourier shell correlation at FSC = 0.5 and was 23.5 Å.

### Surface plasmon resonance

SPR experiments were performed using a Biacore T200 machine (GE Healthcare Life Sciences) at 20°C in 10 mM HEPES (pH 7.5) and 150 mM NaCl. PtdIns(4,5)P_2_ (C-45B6a) and PtdIns(3,4,5)P_3_ (C-39B6a) were purchased from Echelon Biosciences. The BIAcore CM5 chip (GE Healthcare Life Sciences) was firstly covered with streptavidin following the manufacturer's instructions before the biotinylated PIPs or biotinylated proteins were immobilised. The analyte with twofold serial dilutions was applied at a flow rate of 20 µl/min for 180 s followed by 600 s of dissociation time. The biosensor chip was regenerated after each sample injection cycle with a different buffer in the two experimental set-ups: in the case of immobilised PIPs on the sensor chip, 0.1% SDS was used; when the PH domain was immobilised, then the surface was regenerated by 2 M MgCl_2_. The data were fit with a 1:1 Langmuir adsorption model (*B* = *B*_max_*C*/(*K*_D_ + *C*), where *B* is the response of bound analyte and *C* is the concentration of the analyte in the sample to calculate the dissociation constant (*K*_D_) using the BIAanalysis software. The molecular mass of the nanodiscs for SPR analysis was estimated to be 120 kDa.

### Molecular dynamics simulations

#### CG-MD simulations

The CG-MD simulations were performed using GROMACS 4.5.5 [64] with the Martini 2.1 force field [65,66]. The simulation set-up is shown in Figure 2. A summary of all simulations performed is present in Table 2. Prior to the production simulations, all systems were energy minimised and then equilibrated for 500 ns with the protein backbone particles restrained. For each repeat simulation within an ensemble, the protein was rotated around the *x*, *y*, and *z* axes to form a different initial configuration. An ensemble of 20 simulations of 1.5 µs each was run with a time step of 20 fs. An elastic network model was applied to all backbone particles with a cut-off distance of 0.7 nm [67]. The LINCS algorithm was used to constrain to equilibrium bond lengths [68]. Lennard–Jones interactions were shifted to zero between 0.9 and 1.2 nm. Coulombic interactions were shifted to zero between 0 and 1.2 nm. The pressure was 1 bar and the temperature was 323 K. A Berendsen's algorithm [69] was used to control the pressure and the temperature with a coupling time of 1 ps.

#### AT-MD simulations

Conversion of CG to atomistic systems was made using a fragment-based approach [70]. The snapshot that was converted into an atomistic representation corresponded to the preferred orientation of the kindlin-3 PH domain in the density landscapes shown in Supplementary Figure S5. The GROMOS96 43a1 force field [71] was used with SPC water molecules. The temperature was 323 K. The velocity rescaling method [72] was used to control the temperature with a coupling time of 0.1 ps. The pressure was 1 bar, and it was controlled with semi-isotropic pressure coupling using the Parrinello–Rahman barostat [73] with a coupling time of 1 ps. Bond lengths were constrained to equilibrium lengths using the LINCS method. The time step was set at 2 fs. The particle mesh Ewald method was used to model the electrostatic interactions. For the atomistic simulation with the kindlin-3 PH domain in solution, both the GROMOS96 43a1 and the OPLS-AA force field within GROMACS were used. For these simulations, an isotropic pressure coupling was used. The temperature was 310 K.

#### PMF simulations

Initial structures of WT or mutant kindlin-3 PH domain bound to PIP_3_ or PIP_2_ in a 80:20 POPC:POPS bilayer were generated by alignment with an existing Grp1–PH/PIP_3_ complex (see ref. [34]). The system was converted into a coarse-grained representation. The PH domain was pulled from the bound PIP lipid along the membrane normal (*z*-axis) by fixing the centre of mass to a reference point, which was moved at a rate of 0.001 nm/ps for a total distance of 2.6 nm, while the PIP phosphate atom was restrained to its initial position. Snapshots with protein–lipid separations (measured from the PH domain centre of mass to the phosphate atom of the PIP) in 0.1 nm intervals for the first 2 and 0.2 nm thereafter were extracted and used as initial structures for umbrella sampling simulations. In each simulation, the phosphate atom was again restrained to its initial position and the *z*-distance of the PH centre of mass was restrained relative to the phosphate atom with a force constant of 1000 kJ/mol/nm^2^. To reduce the time required for adequate sampling, the PH domain centre of mass was also restrained in the *x* and *y* directions, by a force constant of 100 kJ/mol/nm. Each window was simulated for 1 µs (mutant), 1.4 µs (WT/PIP_3_), or 1.5 µs (WT/PIP_2_), with the distance between the PH centre of mass and the PIP atom bead along the *z*-axis recorded every 20 ps. PMF profiles were calculated using the GROMACS *g_wham* tool [74]. The time taken to reach convergence was judged from profiles generated for sequential time intervals; data before this time were discarded as equilibration before a final profile was calculated. Errors were obtained from bootstrap analysis. All profiles were shifted, so the value of the PMF in bulk was 0 kcal/mol.

## Abbreviations

AT-MD: atomistic molecular dynamics
CG-MD: coarse-grained molecular dynamics
EMT: epithelial-to-mesenchymal transition
FERM domain: 4.1-erythrin–radixin–moiesin domain
FSC: Fourier shell correlation
Grp1: general receptor of phosphoinositide 1
GST: glutathione *S*-transferase
IPRR: isoleucine–proline–arginine–arginine
LAD-III: leucocyte adhesion deficiency type III
MD: molecular dynamics
MR: molecular replacement
PH: pleckstrin homology
PMF: potential of mean force
POPS: palmitoyl-oleoyl glycerophosphoserine
PtdInsP/PIP: phosphatidylinositol phosphate
RMSD: root mean square deviation
SPR: surface plasmon resonance
WT: wild type.
